# Changes in dimension of neurovascular canals in the mandible and maxilla: A radiographic finding in patients diagnosed with MRONJ

**DOI:** 10.4317/medoral.22274

**Published:** 2018-04-24

**Authors:** Duygu Goller-Bulut, Gözde Özcan, Fatma Avci

**Affiliations:** 1 Assistant Professor, Department of Oral and Maxillofacial Radiology, Faculty of Dentistry, Abant İzzet Baysal University, Bolu, Turkey; 2 Specialist Dentist, Department of Oral and Maxillofacial Radiology, Turkey; 3 Research Assistant, Department of Oral and Maxillofacial Radiology, Faculty of Dentistry, Erciyes University, Kayseri, Turkey

## Abstract

**Background:**

The aim of this retrospective study was to compare the morphological features of neurovascular canals and foramina of patients with medication-related osteonecrosis of the jaws (MRONJ) and healthy individuals by using cone beam computed tomography (CBCT).

**Material and Methods:**

The CBCT images of 58 patients under bisphosphonate therapy diagnosed with MRONJ and age gender- matched controls were retrospectively evaluated. The diameter of mandibular and nasopalatine canal and mandibular, mental and lingual foramina were measured on several sections of CBCT. The value of mental index (MI) and panoramic mandibular index (PMI) were also assessed.

**Results:**

The mean value of diametric measurements for all neurovascular canals and foramina in MRONJ patients were narrower than controls. Left mandibular foramen was the most affected area (*p*<0.001). There were significantly difference in all measurements of mental foramen, lingual foramen and mandibular incisive canal between two groups (*p*<0.05). PMI of MRONJ subjects were also significantly differences in both sides (*p*<0.05).

**Conclusions:**

In MRONJ patient, neurovascular canals and foramina are affected due to the alterations in bone remodeling. Therefore, the diametric measurement of neurovascular canals and assessment of MI and PMI on CBCT, is a potentially useful method for detection of early changes associated with bisphosphonate therapy and for predict areas where new necrosis may occur.

** Key words:**Bisphosphonate, MRONJ, CBCT, neurovascular canals, PMI.

## Introduction

Medication-related osteonecrosis of the jaw (MRONJ) is a quite serious drug reaction, consisting of bone destruction in the maxillofacial region of patients. Osteonecrosis can be caused by two pharmacological agents: Antiangiogenic and antiresorptive drugs including bisphosphonates (BPs) receptor activator of nuclear factor kappa-B ligand inhibitors (1). Our study group was consisted of BPs medicated patients. BPs are the first-choice antiresorptive drugs of management for diseases as osteoporosis, multiple myeloma, metastatic bone cancer, hypercalcemia related to malignancy and Paget’s (1). They are inorganic pyrophosphates effective in inhibiting osteoclast-mediated bone resorption (2). MRONJ is a severe complication of BP medication. Etiopathogenic mechanisms of MRONJ have been poorly understood. It generally occurs after a secondary infection or minor local trauma during a dental treatment, but no work can clarify all morphological changes observed at the microscopic and macroscopic level (3).

The formation of BPs medication related osteonecrosis is related to long-term treatment with high levels of BPs and was first described in 2003 (4). According to the American Association of Oral and Maxillofacial Surgeons (5). MRONJ is described by the presence of exposed bone in the maxillofacial area more than 8 weeks, an anamnesis of present or prior treatment with BPs and the exception of a radiation therapy history to the maxillofacial region. It may remain asymptomatic for months, hence Stage 0 was defined as a level of potentially emerging MRONJ in line with the clinical guideline (5). It is associated with a high morbidity, causing tooth mobility, pain, paraesthesia, halitosis, bone sequestrum formation and extraoral or intraoral fistula (6).

MRONJ is diagnosed based on clinical features but imaging is crucial for detecting early stages of disease. Although, various radiological techniques have been applied in MRONJ cases, conventional radiography and CT scans are commonly used. In recent years, cone-beam CT (CBCT) has been presented as a diagnostic device using cone-beam geometry, flat panel detectors and 3D reconstruction algorithms that has lower doses and costs less than conventional CT (7).

The CBCT images are not only used as a tool for the measurement of morphological differences, but also are utilized for providing beneficial data to analyze even minor osseous differences. Therefore, CBCT can investigate sclerotic changes in the spongious bone of the jaw, non-healing extraction sockets and a thickened lamina dura as characteristic findings under medication with BP in individuals regarded as risk for MRONJ, even without the presence of a sequestrum or other clinical symptoms (6).

This study was aimed to investigate the changing in dimension of neurovascular canals, mental index (MI) and panoramic mandibular ındex (PMI) by using CBCT that may help to understand whether it is a diagnostic value in early diagnosis of MRONJ.

## Material and Methods

- Study Group

The study group consisted by fifty-eight patients (26 females, 32 males, mean age 70.3 years; age range 49–84 years) under bisphosphonate therapy were retrospectively included between April 2012 and December 2016. Inclusion criteria were overt MRONJ diagnosed by a maxillofacial surgeon with 7 years of professional experience based on generally accepted diagnostic criteria of the clinical introduction (5). Patient with MRONJ in stage 1 and 2 (Stage 1: necrotic bone exposure in asymptomatic patients with no evidence of soft-tissue infection; Stage 2: necrotic bone exposure associated with soft-tissue infection and pain) were included in study group (6). Patients with osteonecrosis that affect neurovascular structures in both sides were excluded. In study group all patients had MRONJ in one side of mandibular molar and angulus region.

CBCT images of patients with MRONJ had been archived in Department of Oral and Maxillofacial Radiology, Faculty of Dentistry, Erciyes University. Control group was selected gender- and age-matched healthy individuals who had CBCT images performed for the treatment planning of implant-based prosthesis from the same archives (26 females, 32 males, mean age 70.2 years; age range 51–82 years). The measurements were made on the same side as the patient group.

Owing to the retrospective nature of this study, ethical board approval was not required by local laws and regulations, and the informed consent requirement was waived by the departments where the study was carried out. The study was performed according to the guidelines of the Declaration of Helsinki concerning Ethical Principles for Medical Research Involving Human subjects.

- Imaging Procedures

All study subjects underwent imaging using the New Tom VG (Quantitative Radiology, Verona, Italy) with flat-panel detector-based cone-beam computed tomography (FPD-CBCT). The X-ray parameters (kV, mA) were automatically determined from scout views by the NewTom VG. All images were obtained with the patient in the supine position. Scanning time was 18 seconds, collimation height was 13 cm, exposure time was 3.6 seconds, and voxel size was 0.3 mm3. Digital Imaging and Communications in Medicine (DICOM) files obtained from the CBCT scans were reconstructed using NNT viewer software. Acquired 3D data were performed by two independent readers who were blinded to each other and patient data (two radiologist with 4 and 2 years of experience in head-and-neck imaging).

- Measurements

**Figure 1:**
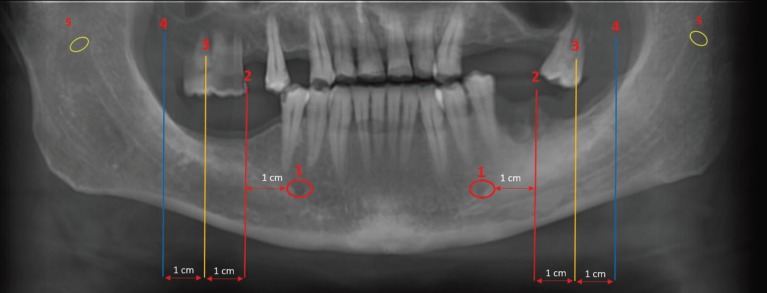
The reconstructed panoramic images showed as follow, 1; mental foramina, 2-4; the measured sections through the mandibular canals, 5; mandibular foramina.

**Figure 2:**
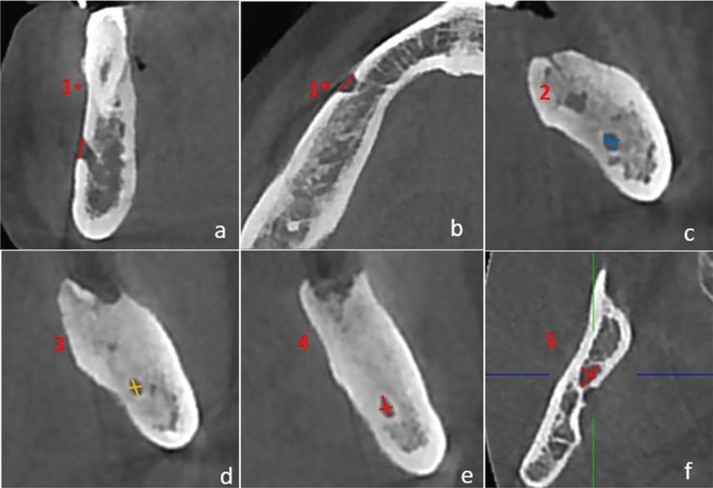
The demonstration of measurements on CBCT images. a. section 1+; cross-sectional measurements of mental foramen, b. section 1*; axial measurements of mental foramen. c-e. The minimum and maximum diametric measurements through mandibular canal on section 2-4. f. The minimum and maximum diametric measurements of mandibular foramen on section 5.

**Figure 3:**
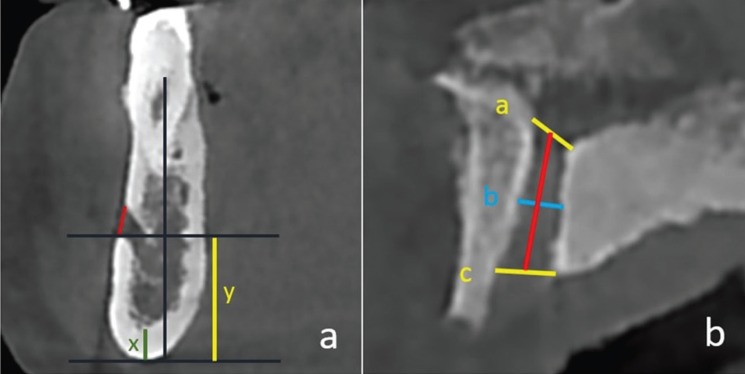
a. x; mental index, x/y; panoramic mandibular index, b. The measurements of nasopalatine canal line 1;nasopalatine foramen, line 2; centre of canal, line 3; incisive foramen.

**Figure 4:**
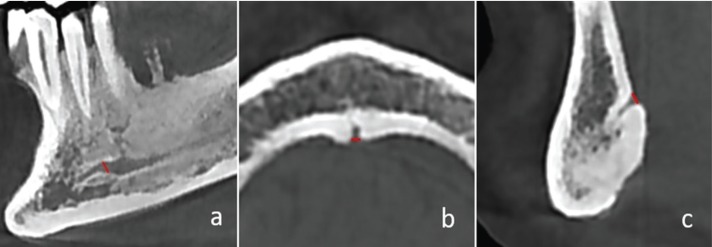
a. The measurements of origin of mandibular incisive canal. b Axial measurements of lingual foramen. c. Sagittal measurements of lingual foramen.

Jaw was divided to 5 sections and all measured sections were shown in Figure 1, on panoramic view. On CBCT scans obtained from MRONJ patients and healthy controls the measurements were performed as follows: diameter of mental foramen (MenF) on axial and sagittal slices (Fig. 2 a,b), on cross-sectional views; three cross-section images with 1 cm intervals of mandibular canal (ManC) and mandibular foramen (ManF) were measured, bilaterally (Fig. 2c-f). The diametric measurements for ManF and ManC were done at both minimum and maximum diameter on cross- section images. For MI, mandibular cortical thickness was measured on the line, which was perpendicular to the bottom of the mandible at the middle of the mental foramen. The PMI was the ratio of the thickness of the mandibular cortex to the distance between the mental foramen and the inferior mandibular cortex (Fig. 3a). On sagittal section, the diametric evaluation of nasopalatine canal (NPC) was assessed on nasopalatine foramen (NPF), incisive foramen (IF) and center of NPC (Fig. 3b). In addition, the origin of mandibular incisive canal (MIC) was evaluated on sagittal images (Fig. 4a). The measurement of lingual foramen (LF) were performed on sagittal and axial sections (Fig. 4 b,c).- Statistical analyses.

Two oral radiologist were carried out all the measurements independently and inter-observer reliability showed a high correlation. The measurements of the first observer with 4 years of experience were used for further analysis. Statistical analyses were conducted with SPSS for Windows SPSS® v. 16.0 (IBM Corp., New York, NY; formerly SPSS Inc., Chicago, IL). The measurments were evaluated using the independent and paired samples t-test to compare the means of the all values between MRONJ and healthy sites and controls. Values of *P*<0.05 were considered to indicate statistical significance.

## Results

58 patients with MRONJ, osteonecrosis were found in 24 right and 34 left sides of the mandible ( Table 1). However, the 24 right and 34 left sides of the 58 healthy individuals with no systemic disorder or medication were comprised in control group.

**Table 1:**
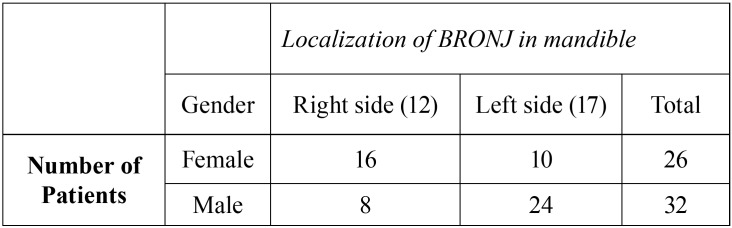
The distribution of patients with medication-related osteonecrosis of the jaw (MRONJ) according to gender.

All comparisons were performed reciprocally between both sides of patients with MRONJ and age-gender matched healthy individuals, expect non-measurable sites due to osteonecrosis. The evaluation was indicated narrowing in neurovascular canals of patients with MRONJ, relatively ( Table 2). All the minimum diameter comparisons of the measurements of MenF in both sides were showed significantly differences between MRONJ subjects and controls (*p*<0.05). ManF and ManC were found statistically significant differences in both sides (*p*<0.05), expect first part of left ManC (*p*=0.45). The most contraction was observed in minimum diameter of left ManF (*p*<0.0001). Among the maximum diametric measurements, the significantly difference was found only at first part of right ManF (*p*=0.02).

**Table 2:**
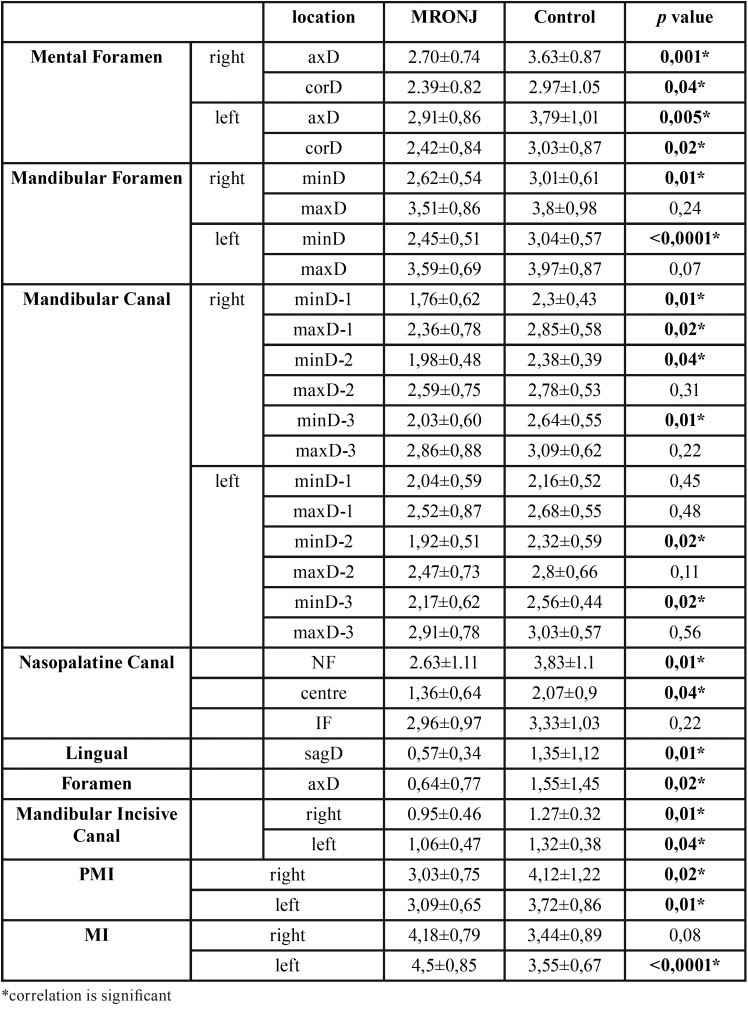
The mean value of neurovascular structures’ diameter, MI and PMI and also p value of comparisons between MRONJ subjects and controls. D; diameter, sag; sagittal, ax; axial, min; minumum, max; maximum.

NPC comparisons showed that there were statistically significant differences in diameters of NPF and center area (*p*=0.01, *p*=0.04, respectively). Although the mean value of IF diameter in MRONJ subjects were narrower than controls, there were no significantly difference between two groups (*p*=0.22). There were significantly differences in sagittal (*p*=0.01) and axial (*p*=0.02) diameters of LF and origin diameters of MIC on both sides (*p*<0.05).

There were significantly differences in both sides of PMI between patients with MRONJ and control group (*p*<0.05). But while, MI evaluation in left side was found significantly difference between two groups (*p*<0.0001), there were no significantly differences for the right sides of subjects (*p*=0.08).

In patient group with MRONJ, because of osteonecrosis we couldn’t make measurements bilaterally except MIC, MenF, ManF, MI and PMI. There are no significant differences for MIC, MenF and ManF, but significantly differences were found for MI and PMI between the osteonecrosed and healthy sides of patients with MRONJ (*p*<0.05) ( Table 3) The mean values of PMI and MI were higher in MRONJ side. No significantly differences were found for all measurements on right and left sides of control group.

**Table 3:**
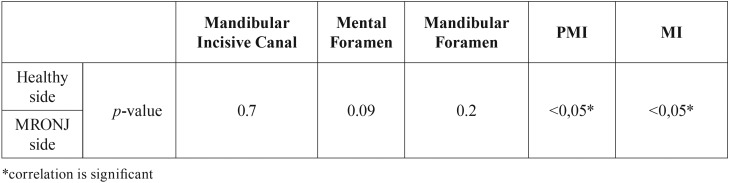
The comparison of MRONJ side and healthy side of MRONJ patients.

## Discussion

The action mechanism of BPs in bone metabolism is not clarified. Due to BPs’ specific affinity to bone, they affect almost only on osseous tissue when applied at physiological doses; thus they accumulate not only in newly formed bone but also in vicinity to the osteoclasts. BPs also decrease bone blood flow and significantly reduce circulating levels of vascular endothelial growth factor, which is essential in the angiogenesis process (8). Furthermore, Miglioratti (9) and Marx (10) have proposed that owing to their antiangiogenic effects, BPs may be directly in charge of MRONJ. MRONJ typically develops after injuries of the mucosa or simple dentoalveolar surgery, or is associated with inadequately well-fitting prostheses. Some papers had also been reported spontaneous cases (11-13).

BPs have specific affinity to bone like maxillary and mandible bones (8). Especially the periodontal and alveolar bone surfaces have high BP uptake and accumulation. They also have relatively high rates of bone remodeling so these regions are more affected. Maxillofacial region is exposed to the external situation through periodontal ligaments and teeth, this may be a probable cause of MRONJ only in the jaw (10,14). BPs show strong anti-osteoclastic activity (8). This may cause uncontrolled bone accumulation in bone surfaces of nourovascular canals and foramens.

Currently, it was supposed that BP uptake, which is in direct relationship to the local proportion of bone turnover, would actually be (approximately 100 times) higher in alveolar bone than in other skeletal sites (15,16). On account of these reasons, finding an early indicator of osseous changes in jaw may enable to understand before developing MRONJ. MRONJ at Stage 0 are defined a great risk of developing severity stages (17,18). Thus, it is critical to diagnose the early silent (asymptomatic) stage of MRONJ. Radiological bone changes specifying possibility of MRONJ might help dentists with preventive approach and treatment planning for patients under BP therapy.

Many osseous radiographic features related to MRONJ in panoramic and intraoral radiographies, computed tomography, magnetic resonance imaging, and positron emission tomography have been designated (2,19-23). Recently, CBCT is considered the most suitable imaging modality for MRONJ, because of its sufficient 3D data for osseous structure, lower radiation doses and attainable cost. In the literature, the radiological findings of MRONJ lesions in CBCT were described as follow, increase in sclerotic expressions, failure of post-surgical remodelling, erosion on the cortical bone and subperiosteal bone deposition which are the most common and most characteristic features of MRONJ (2,24). Nevertheless, these radiographic findings seem useful for predicting that there is a potential early stage of MRONJ, there is no study about alterations in neurovascular structures of MRONJ patients’ jaw. This study was planned to achieve initial findings of MRONJ by diametric measurements of jaws’ neurovascular canals and foramina on CBCT.

Guggenberger *et al.* proved the benefits of CBCT in diagnosis MRONJ by qualitative and quantitative image parameters (1). Torres *et al.* used CBCT for evaluation of fractal dimension and mandibular cortical bone to diagnose early features of MRONJ (7,25). They found significantly differences in both study parameters between patients with BP medication and controls. Phal *et al.* also reported the sclerotic change encroached on the mandibular canal in 3 subjects of 15 patients with MRONJ (20). Based on the results of these studies, we thought that subperiostal bone accumulation may cause the changing on dimension of neurovascular constitution. The results of current study are supported to our opinion that the narrowing in neurovascular structure of patients with MRONJ is substantially.

The MI and PMI were utilized for assessment of differences between various patient groups in several studies. In this study, there were significantly difference in indexes between groups (*p*<0.05, except MI of right side). Furthermore, the results showed the most affected area is MenF whose all measurements’ were significantly differences between two groups. The diameter of LF was also found substantially narrowed in patients with MRONJ. The NPC, NPF, MIC and minimum diameter of ManF and ManC, which were significantly narrower in MRONJ subjects, may have a diagnostic value when display only some of them in small field of view (FOV). As a result, the present study suggested that these diametric measurements and indexes are also useful tools for MRONJ risk evaluaion in BPs-exposed patients.

Due to nature of the retrospective study, present study was limited to the cases of MRONJ that were archived at the Department of Oral and Maxillofacial Radiology at the University of Erciyes, the main limitation of this study was the fact that the bisphosphonates type, underlying disease, dose and duration of bisphosphonate therapy were not known. The risk for MRONJ has been indicated to be dependent on time, dose and type of bisphosphonates. But our parameters which were performed in this retrospective study, are not affected by these clinical informations.

## Conclusions

As known that an external effect is responsible for formation of MRONJ almost everytime, so the preventive approach is crucial for dentists. However, owing to the difference in neurovascular canal and foramen sizes, PMI and MI, CBCT evaluation of patients under bisphosphonates theraphy before surgical procedures could be able to demonstrate the osseos changes of the jaws that may change the treatment plan according to bone conditions. Changes in dimension of neurovascular canals may be an indicator for early diagnosis, but in order to say that we would have to follow subjects when they start are on bisphosphonates see if the changes are a predictor of or early indicator of MRONJ in those who develop the condition.
